# Treatment of non-high-risk acute promyelocytic leukemia with realgar-indigo naturalis formula (RIF) and all-trans retinoid acid (ATRA): study protocol for a randomized controlled trial

**DOI:** 10.1186/s13063-019-3983-2

**Published:** 2020-01-02

**Authors:** Xinxin Zhang, Li Liu, Yazhou Yao, Sha Gong, Mengchang Wang, Jieying Xi, Limei Chen, Suhua Wei, Huiyun Zhang, Chenyang Zhao, Huaiyu Wang

**Affiliations:** 1 Department of hematology, First affiliated hospital of Xi’an Jiaotong University, Yanta West Road, Xi’an, 710061 Shaanxi China; 2 Department of hematology, Tangdu Hospital, Fourth Military Medical University of Chinese PLA, No. 1 Xinsi Road, Xi’an, 710038 Shaanxi China; 3 Department of hematology, Baoji Central Hospital, jiangtan road, Baoji, 721008 Shaanxi China

**Keywords:** Acute promyelocytic leukemia, Oral realgar-indigo naturalis formula, Arsenic trioxide, All-trans retinoic acid

## Abstract

**Background:**

Acute promyelocytic leukemia (APL) is a highly curable disease when treated with all-trans retinoid acid (ATRA) and arsenic trioxide (ATO). The combination of ATO and ATRA has become the standard therapeutic protocol for induction therapy in non-high-risk APL. An oral arsenic realgar-indigo naturalis formula (RIF) has also showed high efficacy and it has a more convenient route of administration than the standard intravenous regimen. Unlike in previous trials, the arsenical agent was used simultaneously with ATRA during post-remission therapy in this trial.

**Methods:**

This study was designed as a multicenter, randomized controlled trial. The trial has a non-inferiority design with superiority being explored if non-inferiority is identified. All patients receive ATRA-ATO during the induction therapy. After achieving hematologic complete remission (HCR), patients were randomly assigned (1:1) to receive treatment with ATRA-RIF (experimental group) or ATRA-ATO (control group) as the consolidation therapy. During the consolidation therapy, the two groups receive ATRA plus RIF or intravenous ATO 2 weeks on and 2 to ~ 4 weeks off until molecular complete remission (MCR), then ATRA and oral RIF 2 weeks on and 2 to ~ 4 weeks off giving a total of six courses.

**Discussion:**

This trial aims to compare the efficacy of ATRA-ATO versus ATRA-RIF in non-high-risk patients with APL, to demonstrate that oral RIF application reduces the total hospitalization days and medical costs. The simple schedule was studied in this trial.

**Trial registration:**

ClinicalTrials.gov, NCT02899169. Registered on 14 September 2016.

## Background

Acute promyelocytic leukemia (APL) is characterized by a chromosomal translocation of t (15; 17) (q22; q21), involving the retinoic acid receptor alpha (RARα) and promyelocytic leukemia (PML) [1]. Before the use of all-trans retinoid acid (ATRA) APL was a highly fatal disease because of potentially severe coagulopathy [2]. Recent clinical trials have demonstrated that high complete remission (CR) rates and disease-free survival (DFS) can be achieved using a combination of ATRA and arsenic trioxide (ATO) as a first-line treatment for newly diagnosed APL [3–6].

Moreover, the GIMEMA group demonstrated broadly better quality of life (QoL) outcomes post-induction, favoring treatment with intravenous ATO compared to standard chemotherapy [7]. ATRA combined with oral realgar-indigo naturalis formula (RIF) has been shown to reduce the medical costs and length of hospital stay during induction and remission therapy compared with ATO plus ATRA in patients with APL [8]. Based on these clinical trials, compared with intravenous ATO, oral RIF may achieve comparable remission and survival outcomes at a lower cost and with a shorter hospital stay when used as the first-line treatment in APL.

Different arsenical agents were used in previous trials, such as oral RIF, tetra-arsenic tetra-sulfide [9] or intravenous ATO, and ATO was also used in different doses and schedules. For example, ATO was used at 0.15 mg/kg body weight/day for 5 days/week for 4 weeks every 8 weeks in study APL0406 [4], but in study AML17 [10], ATO was used at 0.25 mg/kg body weight/day twice a week in post-remission therapy. In this randomized, controlled trial, a new protocol was designed with the aim to simplify treatment and to further improve the curative effect.

## Methods

### Study hypothesis

The hypothesis is that ATRA-RIF is not inferior to ATO-ATRA in treating non-high-risk APL. Application of oral RIF may reduce the total hospitalization days and medical costs. The simultaneous application of oral RIF and ATRA is more convenient for patients and improves the efficacy.

### Eligibility criteria

Patients will be enrolled from the following hospitals: The First Affiliated Hospital of Xi’an Jiaotong University and Tang-Du Fourth Military Medical University of Chinese PLA. All study participants will provide signed written informed consent before participation. All participants will go through a standardized interview process and receive more information about the study and the treatments. The purpose, procedures, potential risks and benefits of the study will also be explained thoroughly to the participants. The participants will be able to withdraw from the study at any time without consequence.

### Inclusion criteria

Participants meeting the following criteria will be included:

- Age 14–75 years
- Newly diagnosed APL with t (15; 17) (q22; q12)
- Pre-treatment peripheral blood white blood cell (WBC) count < 10 × 10^9^/L
- Able to complete the entire treatment process
- Signed written informed consent provided by the patient or their family

### Exclusion criteria

Participants meeting one or more of the following criteria will be excluded:

- Allergy to the drug ingredient or the supplementary material, or having an allergic constitution
- Cardiac insufficiency (cardiac contraindications to intensive chemotherapy (left-ventricular ejection fraction (L-VEF) < 50%)).
- Inadequate renal reserve (defined as total creatinine > 3 times the institutional upper limit of the normal range)
- Significant arrhythmias, electrocardiogram (EKG) abnormalities (congenital long QT syndrome; history or presence of significant ventricular or atrial tachyarrhythmia; clinically significant resting bradycardia (< 50 beats per minute); QTc > 450 ms on screening EKG; right bundle branch block plus left anterior hemiblock, bifascicular block)
- Combined with other malignant tumors
- Pregnant and lactating women
- Participants in other drug trials in the last 3 months
- Suffering from mental illness or other circumstances which render them unable to complete the treatment plan

### Sample size calculation

On the basis of the previous studies [4, 9, 11] assuming 97% DFS in the control (ATO) group, and conservatively assuming 99% DFS in the experimental (RIF) group, a noninferiority margin of − 5%, follow up of 2 years, 5% type I error (one-sided), 90% power, we used PASS 11 to calculate a required sample size of 53 evaluable patients per group to draw a conclusion of non-inferiority. Allowing for a withdrawal rate of 10%, 59 patients per group will be required. Therefore, we plan to enroll 118 patients into two groups (1:1). We plan a final analysis only, with no interim analysis. The design of this trial is a non-inferiority trial with superiority being explored if non-inferiority is identified.

### Randomization

**Fig. 1:**
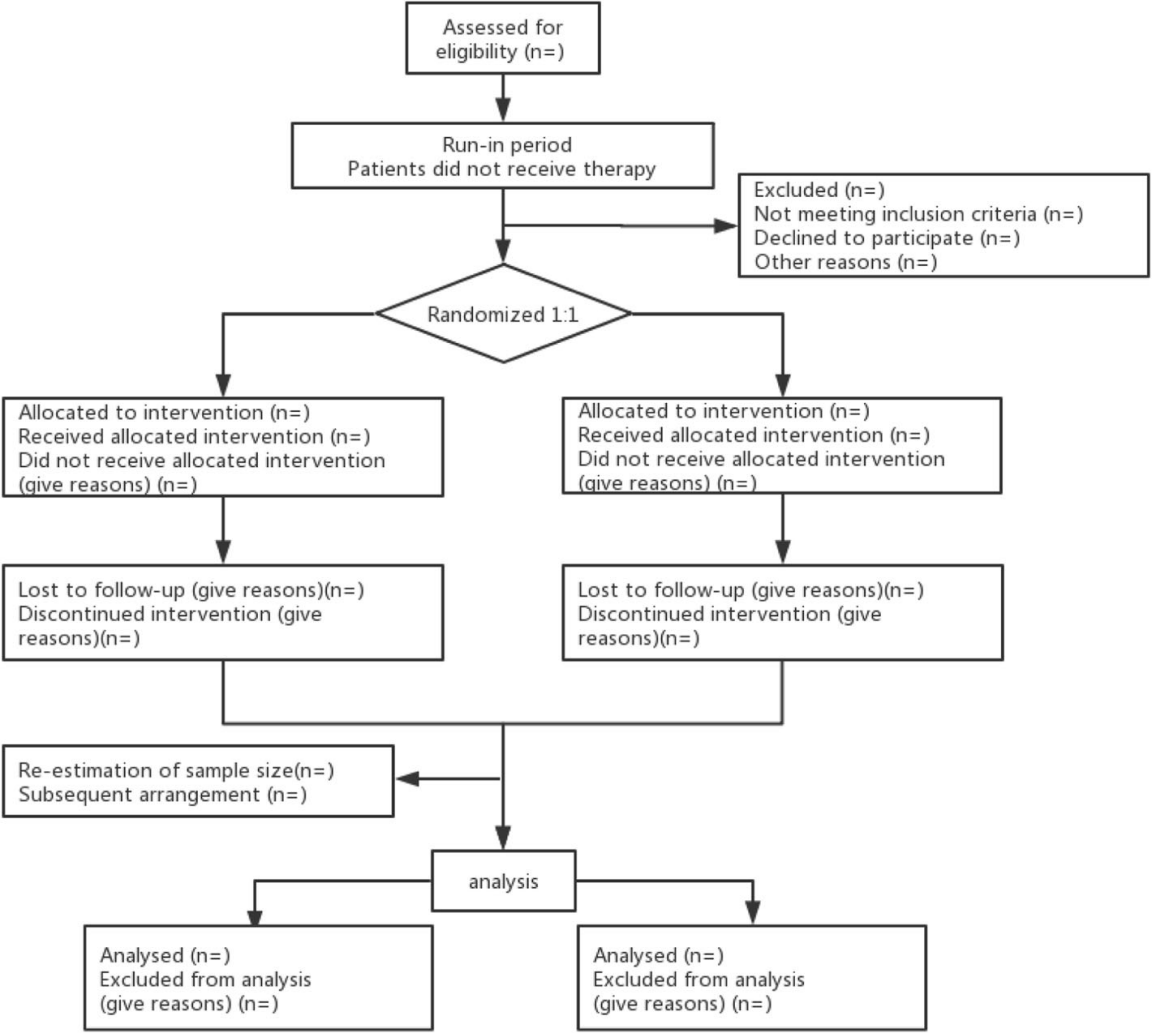
Trial flow

**Fig. 2:**
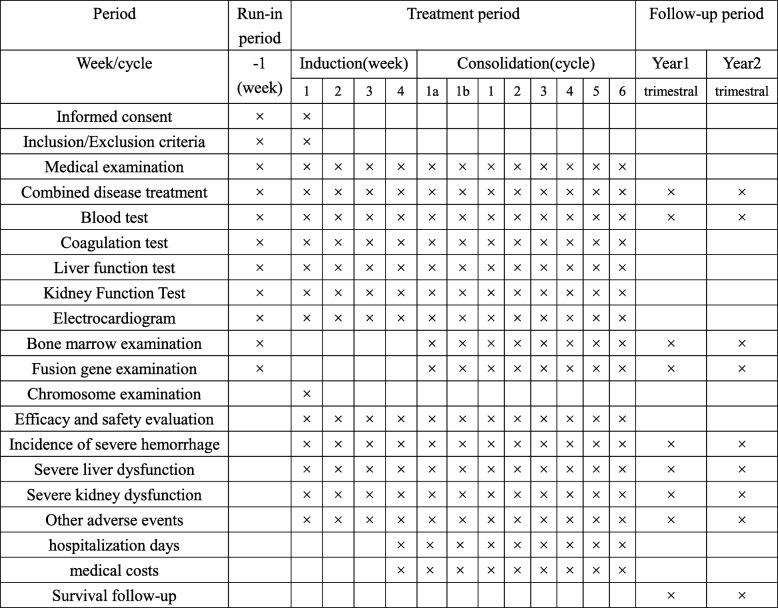
Trial process

Treatment allocation occurs when the study participant meets the inclusion criteria and signs the informed consent form. All eligible patients will receive ATRA-ATO for induction therapy. Participants are randomized by permuted blocks to ensure there are similar numbers of participants in the intervention and control groups. The randomization allocation schedule is generated by a statistician with no other involvement in the study. Once consented, participants are allocated in a 1:1 ratio to the two arms of the study according to a computer-generated random sequence after achieving hematologic complete remission (HCR) (Figs. 1 and 2). Participants will be informed of their allocated treatment group following randomization.

### Blinding

No blinding method will be used in this experiment. Blinding is not feasible for this study for patients or the research team. The data analyst is also involved directly with the study processes and data collection, and thus blinding of the analyst is not possible.

### Interventions

**Fig. 3:**
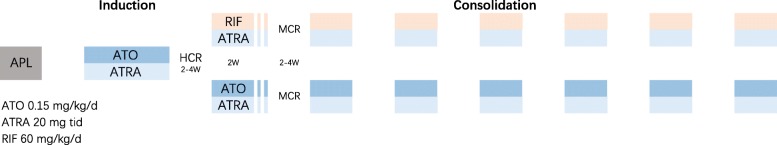
Treatment groups. ATO, arsenic trioxide; ATRA, all-trans retinoic acid; APL, acute promyelocytic leukemia; HCR, hematologic complete remission; MCR, molecular complete remission; RIF, realgar-indigo naturalis formula

Patients with APL will be randomly assigned by a computer-generated, random-allocation schedule to one of two groups: the ATRA-RIF group (experimental group); the ATRA-ATO group (control group); patients with APL will be classified into three risk categories on the basis of WBC count and platelet count. Low risk is a WBC count < 10 × 10^9^/L and a platelet count ≥40 × 10^9^/L; intermediate risk is a WBC count < 10 × 10^9^/L and a platelet count < 40 × 10^9^/L; high risk is a WBC count ≥ 10 × 10^9^/L (Fig. 3).

### Induction therapy

The induction therapy is ATRA 60 mg/day (20–45 mg/m^2^ body surface area/day) plus ATO (0.15 mg/kg body weight/day), which will be maintained until HCR. Hydroxyurea will be used in patients who develop leukocytosis during induction therapy. Four blood tests are performed per week during induction therapy. The blood routine should be tested everyday in the first week of induction therapy.

### Consolidation therapy

#### ATRA-RIF group

The ATRA-RIF group will receive ATRA 60 mg/day (20–45 mg/m^2^/day) plus oral RIF (60 mg/kg/day) 2 weeks on and 2 to ~ 4 weeks off, until PML-retinoic acid receptor alpha (RARα) is negative by reverse transcriptase-polymerase chain reaction (RT-PCR), then ATRA and oral RIF 2 weeks on and 2 to ~ 4 weeks off, giving a total of six courses.

#### ATRA-ATO group

ATRA 60 mg/day (20–45 mg/m^2^/day) plus ATO (0.15 mg/kg/day) 2 weeks on and 2 to ~ 4 weeks off, until PML-RARα is negative by RT-PCR, then ATRA and ATO 2 weeks on and 2 to ~ 4 weeks off, giving a total of six courses.

The PML-RARα fusion gene will be detected by RT-PCR before every cycle of consolidation therapy. The liver and kidney function, blood coagulation, electrocardiogram, arsenic concentration in blood and urine will be checked at least once a week during the whole treatment.

Regarding management of differentiation syndrome (DS) for the two groups: the hydroxyurea should be given at the earliest manifestations of suspected DS and hyperleukocytosis. At the same time, intravenously administered dexamethasone is administered at a dose of 10 mg every 12 h until the disappearance of signs and symptoms for a minimum of 3 days. The ATRA can still be given, but the dose of ATRA should be reduced by as much as half the conventional dose.

### Monitoring

#### Follow-up monitoring

We will monitor bone marrow samples by PCR every 3 months after finishing the consolidation therapy to detect molecular relapse. The sustained observation will last for 2 years. If the PML-RARα gene expression is positive, PCR will be repeated for confirmation within 4 weeks. If the PML-RARα gene expression is negative, the patients need to proceed to consolidation therapy or conversely the patient needs to be treated for relapse.

#### Data monitoring

The study progress, safety data and data quality will be monitored by The Clinical Research Center of the First Affiliated Hospital of Xi’an Jiaotong University.

### Harms

Every case of hyperleukocytosis and QT-interval prolongation occurring during the study must be recorded. The following information will be recorded: occurrence time, severity, duration, adopted measure, and the outcome of the adverse event.

### Baseline characteristics

Baseline characteristics in each group will be analyzed using descriptive statistics, including means or medians for continuous variables and percentages for categorical variables.

### Outcome measures

In this trial, we selected 2-year DFS rates as the primary outcome. Any of the following events will be considered a failure: no achievement of HCR after induction therapy, relapse, or death. HCR is defined as follows [12]: hematologic complete remission: bone marrow blasts < 5%; absence of circulating blasts and blasts with Auer rods; absence of extramedullary disease; absolute neutrophil count > 1.0 × 10^9^/L; platelet count > 100 × 10^9^/L.

#### Relapse is defined as follow [12]:

Hematologic relapse is defined by bone marrow blasts of 5%, reappearance of blasts in the blood, or development of extramedullary disease. Molecular relapse: if studied pre-treatment it is defined by recurrence of minimal residual disease (MRD) as assessed by quantitative RT-qPCR or by multi-color flow cytometry.

The secondary outcomes of this trial are hospitalization days and medical costs in the two arms. The medical costs will be calculated for each patient. The hospitalization costs for induction and consolidation therapy will be calculated for inpatients. The hospital costs for induction therapy and consolidation therapy will be calculated for outpatients. All medical costs can be collected and multiplied by the unit cost of each resource use. After the patient is discharged from the hospital, we can obtain the medical costs from the medical record in hospital according to the patient’s name/case number. For the outpatients, the medical costs can be obtained from the outpatient system after the patient’s visit. Hospitalization days will be recorded over a 10-month time frame.

### Safety and adverse events

Any serious adverse effects that occur in the study period should be recorded. If adverse events occur, the investigator will determine whether the participant should withdraw from the study, according to the condition of the patient. For serious adverse events, the investigator must immediately take necessary measures and report to the investigator and the ethics committee. Serious adverse effects that are still ongoing at the end of the study period must be followed up to determine the final outcome.

### Data collection and management

Patient information will be entered into the paper case report forms (CRFs) promptly and synchronously with input into the electronic CRF. The occurrence of unexpected problems during this process should be recorded, and will be informed in a timely manner.

In order to ascertain the two randomized groups are similar, the demographic/disease characteristics including sex, age, region, body mass index (BMI) and medical history will be collected.

The committee that was responsible for the study design and the implementation process will ensure the effectiveness and integrity of the trial design. The committee will supervise the integrity and accuracy of data collection to control its quality. The committee also needs to evaluate key outcomes based on the clinical expertise of its members.

Data sharing is not applicable to this protocol article as no datasets have as yet been generated or analyzed during the current study. The results should be made public within 24 months of reaching the end of the study. The end of the study is the time point at which the last data items are to be reported, or after the outcomes of data are sufficiently mature for analysis as defined in the statistical analysis section. A full report of the outcomes should be made public no later than 3 years after the end of the study. Results will also be available through the Clinical Trials Registry.

Missing data will be reviewed to identify potential patterns, and examined to assess how these patterns impact our results. There are three types of missing data that can occur when the data are being collected [13]. When data are missing (completely) at random, unbiased results can still be obtained using the maximum likelihood method that will be used in the linear mixed model analysis. When data are not missing at random, we will analyze sensitivity using pattern mixture models and the results will be used to assess the impact of missing data on the reported conclusions.

### Statistical analysis

Statistical analysis will be performed using SPSS software 11.0. The primary analysis will be conducted on the intention-to-treat principle. Descriptive analysis (calculations of averages, frequencies, proportions, or rates) will be conducted. Comparisons will be made using Student’s *t* test or the Mann–Whitney *U* test for comparison of continuous variables and Pearson’s *χ*^2^ test for dichotomous variables. Student’s *t* test will be used to compare variables of normal distribution. The Mann–Whitney *U* test will be used to compare variables of non-normal distribution. The chi-square test or Fisher’s exact test will be used for comparison of categorical variables between the two groups. Survival functions will be estimated using the Kaplan-Meier method and will be compared using the log-rank test. The medical costs will be retrospectively calculated for the patients involved in a prospective randomized controlled trial and will be compared in a post hoc analysis of the two groups. The Wilcoxon matched pairs test will be used to compare the medical costs between the RIF and ATO groups. All statistical tests will be two-sided, and the level of significance will be set at 0.05, except for the non-inferiority hypothesis.

### Standard Protocol Items: Recommendations for Interventional Trials (SPIRIT)

This protocol has been written in accordance with the SPIRIT guidelines. The SPIRIT checklist is in Additional file 1.

## Discussion

Due to the introduction of ATRA and ATO, the outcome of patients with APL has dramatically improved over the last two decades. The APL0406 study showed the advantages of ATRA-ATO over ATRA-CHT in non-high-risk APL. In a clinical trial in China, a home-based treatment protocol with oral RIF plus ATRA was shown to be effective [11]. Medical costs and numbers of hospital days will be reduced [8]. However, the dosing schedules use of for arsenical agents were different in previous trials. Unlike in the APL0406 study, here, the arsenical agents and ATRA were used simultaneously for 2 weeks every 4 weeks. The simultaneous application of oral RIF and ATRA is more convenient for patients and may improve the efficacy [14].

The peak plasma arsenic after intravenous ATO may be several times greater than that of oral arsenical agents [15]. So, the patients may achieve molecular complete remission (MCR) quickly with ATO. In our trial, all the patients with APL will be treated with a combination of ATRA plus ATO during induction therapy.

Although the time to achieve HCR is about 30 days [16], the time to achieve MCR is different. In a clinical trial, the time to MCR ranges from 2 to 6 months [17]. However, in the recent major clinical trials, all of the patients received the same therapeutic courses in consolidation without considering the time to MCR. In this trial, patients will receive a total of six courses of ATRA-RIF or ATRA-ATO therapy after their PML-RARα is negative, which may result in a lower relapse rate.

Oral RIF is commercialized and commonly available in China. During remission therapy, the patients in the RIF group will be treated at home, while the patients in the ATO group will be treated in the hospital. Compared to intravenous ATO, RIF is relatively inexpensive and can be administered orally in outpatients. Thus, oral RIF may decrease the length of hospital stay and medical costs associated with APL treatment.

## Trial status

Recruitment commenced in July 2016 and the trial is scheduled to end in May 2020.

## Supplementary information

**Additional file 1.** Standard Protocol Items: Recommendations for Interventional Trials (SPIRIT) 2013 checklist.

## Abbreviations

APL: Acute promyelocytic leukemia
ATO: Arsenic trioxide
ATRA: All-trans retinoic acid
CI: Confidence interval
CRF: Case report form
DFS: Disease-free survival
DS: differentiation syndrome
HCR: Hematologic complete remission
MCR: Molecular complete remission
PML: Promyelocytic leukemia
QoL: Quality of life
RARα: Retinoic acid receptor alpha
RARα: Retinoic acid receptor alpha
RIF: Realgar-indigo naturalis formula
RT-PCR: Reverse transcriptase-polymerase chain reaction
